# Artificial Grain Boundaries Created by Freestanding Membrane Transfer Reveal Angle‐Dependent Ferroelectric Switching and Leakage in BaTiO_3_

**DOI:** 10.1002/advs.77239

**Published:** 2026-10-14

**Authors:** Shin‐Rou Yin, Yu‐Hsin Lin, Yun‐Guan Wei, Yu‐Cheng Fang, Yung‐Chi Su, Yu‐Wei Chen, Yu‐Ting Lin, Chia‐Chun Wei, Chih‐Hung Wu, Ching‐Yu Chiang, Heng‐Jui Liu, Jan‐Chi Yang, Yi‐Chun Chen, Yen‐Lin Huang

**Affiliations:** ^1^ Department of Materials Science and Engineering National Yang Ming Chiao Tung University Hsinchu Taiwan; ^2^ Department of Physics National Cheng Kung University Tainan Taiwan; ^3^ Department of Materials Science and Engineering National Chung Hsing University Taichung Taiwan; ^4^ National Synchrotron Radiation Research Center Hsinchu Taiwan; ^5^ Center for Emergent Functional Matter Science National Yang Ming Chiao Tung University Hsinchu Taiwan

**Keywords:** coercivity, condensed matter physics, crystallite, electrical measurements, ferroelectricity, grain boundary, materials science, misorientation, piezoresponse force microscopy

## Abstract

Grain boundaries (GBs) strongly influence polycrystalline ferroelectrics, but their discrete effects are difficult to isolate because of nanoscale heterogeneity. Here, we use freestanding membrane transfer to fabricate artificial ${\text{BaTiO}}_{3}$ GBs with controlled, experimentally accessible in‐plane misorientation angles (ϕ). Macroscopic electrical measurements show higher coercive voltage and leakage current at the GB region than in the coherent grain region. Piezoresponse force microscopy directly reveals domain‐wall pinning at the boundary, which impedes lateral domain propagation and increases the effective switching barrier. Conductive atomic force microscopy further identifies the GB as a localized high‐conductance pathway, with leakage increasing strongly with, ϕ in agreement with a Read–Shockley‐inspired model. Temperature‐dependent steady‐state DC measurements yield an activation energy of approximately 0.032 eV at the GB, an order of magnitude lower than in the grain interior. Comparison of Schottky and Poole–Frenkel analyses indicates that transport is more consistent with defect‐assisted, Schottky‐like thermally activated emission than with bulk Poole–Frenkel conduction. These results establish a tunable artificial‐GB platform that directly correlates misorientation angle with domain‐wall pinning, coercive voltage, and localized leakage, providing a pathway for grain‐boundary engineering in ferroelectric thin films.

## Graphical abstract

Artificial grain boundaries are created in ${\text{BaTiO}}_{3}$ thin films by freestanding membrane transfer, enabling direct isolation of a single boundary. Local ferroelectric switching and leakage are found to concentrate at the grain boundary, while increasing misorientation systematically enhances conduction. The results reveal how grain‐boundary angle governs defect‐mediated transport and switching in ferroelectric oxides.

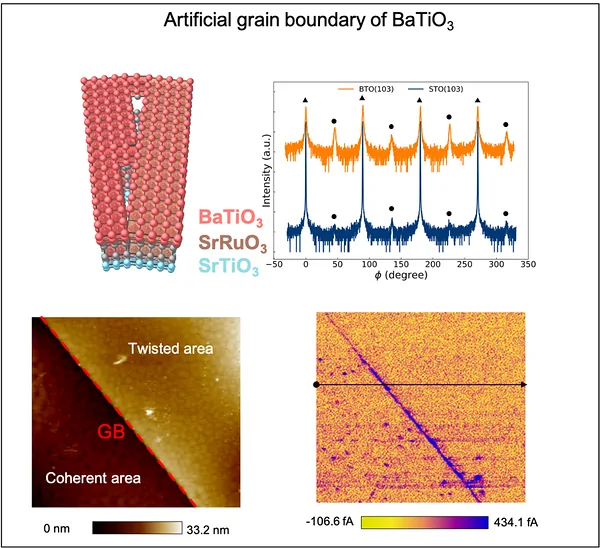

## 1 Introduction

Grain boundaries (GBs) are planar defects that separate neighboring grains in crystalline materials and often exert a disproportionate influence on macroscopic properties. As internal interfaces, GBs frequently serve as sinks for structural and chemical imperfections, including dislocations, strain fields, and point defects such as oxygen vacancies [1, 2, 3]. These local discontinuities can markedly alter the electronic structure, charge transport, and dielectric response relative to the grain interior [4]. In ferroelectric materials, such effects are especially important because polarization switching is highly sensitive to the local electrostatic environment, elastic state, and defect distribution [5, 6]. Since many technologically relevant ferroelectric thin films are polycrystalline and are commonly prepared by deposition methods such as atomic layer deposition, chemical vapor deposition, and sputtering, GBs are unavoidable and may critically influence functional performance [7, 8, 9]. Despite their importance, however, the role of GBs in ferroelectrics remains difficult to resolve because their structural and electrical behaviors are highly localized, spatially heterogeneous, and challenging to probe directly. Therefore, clarifying the role of GBs is essential for both the fundamental understanding and practical optimization of ferroelectric materials.

In polycrystalline ferroelectrics, GBs typically separate grains with characteristic sizes ranging from a few nanometers to micrometers [10]. This random distribution and nanoscale size make direct isolation of individual GB effects experimentally challenging under bulk measurements. To address this challenge, researchers have used grain‐size‐dependent studies, in which observed variations in dielectric and ferroelectric properties are correlated with mean grain size [11, 12, 13, 14]. As for the domain‐switching dynamics, phase‐field modeling and theoretical approaches have shown that GBs act as domain‐nucleation sites [15, 16] as well as domain wall pinning centers at high grain boundary angles [17]. Experimentally, domain wall pinning and leakage current at GBs have been directly observed using piezoresponse force microscopy (PFM) and conductive atomic force microscopy (c‐AFM). For instance, in ${\mathrm{BiFeO}}_{3}$ bicrystals with a $24^{\circ }$ tilt GB, domain walls are attracted to and immobilized by the boundary, with concurrent enhancement of local conductivity attributed to defect accumulation at the GB [18]. For Pb(${\mathrm{Zr}}_{0.2}$ ${\mathrm{Ti}}_{0.8}$)$O_{3}$ prepared by sol‐gel method, domain imaging by PFM also showed that domain wall pinning is strong and stable at grain boundaries, whereas pinning within the grain interior is comparatively weak and unstable [19]. Other studies utilized Band Excitation PFM to reveal a localized decrease in the nonlinear Rayleigh response, providing direct evidence that grain boundaries act as pinning sites that suppress extrinsic domain wall mobility [20, 21, 22]. The c‐AFM results of ferroelectric ${\mathrm{ErMnO}}_{3}$ demonstrated that GBs exhibit an electronic conductance significantly higher than the grain interior, attributed to the localized accumulation of oxygen vacancies [23].

Despite these advances, systematic experimental studies that isolate the effects of GB misorientation angle and directly correlate them with ferroelectric loops, domain dynamics, and electrical transport properties remain limited. Investigations using commercial bicrystal substrates have provided important insights into domain‐wall pinning and nonlinear local response, but they are constrained by the availability of fixed tilt angles, typically in the range of 10
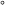
–24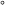, which limits systematic exploration of angle‐dependent GB behavior. To overcome these limitations, we apply a freestanding membrane transfer method [24] to produce artificial BTO grain boundaries with experimentally accessible misorientation angles. This approach enables direct and systematic comparison of the coherent area, twisted area, and grain‐boundary region under otherwise comparable growth and measurement conditions.

Using this platform, we find that GBs lead to higher coercive voltage and enhanced leakage current compared with single‐crystal regions. The increase in coercive voltage is interpreted primarily in terms of propagation‐limited switching caused by GB‐induced domain‐wall pinning, rather than by the nucleation process alone. By systematically varying the GB angle, we observe that leakage current increases with GB misorientation, suggesting that increasing structural disorder at the GB enhances the effective density of transport‐active defect or trap states. Temperature‐dependent leakage measurements further reveal that the grain boundary exhibits a much lower activation barrier than the surrounding regions, indicating that the GB acts as a defect‐rich conduction pathway. In the present work, however, the electrical measurements do not directly identify the microscopic defect species, and therefore oxygen vacancies are discussed only as one possible contributor among several defect types commonly found at oxide grain boundaries.

Such controlled studies deepen our understanding of artificial grain‐boundary physics in ferroelectric oxides and also highlight the unique advantage of the freestanding‐transfer platform over fixed‐angle bicrystal substrates. In particular, the present approach allows direct correlation among GB angle, microscopic domain‐wall pinning, macroscopic coercive‐voltage evolution, and localized leakage transport, thereby providing guidance for grain‐boundary engineering in ferroelectric microelectronics.

## 2 Experimental Methods

The freestanding process is illustrated in Figure 1a. A 10‐nm ${\mathrm{SrTiO}}_{3}$ (STO) membrane was grown on a ${\mathrm{La}}_{0.67}$ ${\mathrm{Sr}}_{0.33}$ ${\mathrm{MnO}}_{3}$ (LSMO) sacrificial layer via pulsed laser deposition (PLD). This LSMO layer was subsequently dissolved in an HCl solution, allowing the freestanding membrane to be transferred onto a new STO substrate. During this process, the final in‐plane crystallographic twist angle (ϕ) was determined during manual transfer and alignment of the ultrathin STO membrane, yielding experimentally accessible artificial GBs in the range of approximately $10^{\circ }$ to $45^{\circ }$. The orientation was verified by STO (103) phi scans (Figure 1b), where high‐intensity peaks (triangles) and low‐intensity peaks (circles) correspond to the STO substrate (SUB‐STO) and the freestanding STO (FS‐STO), respectively. Complete phi scans for all investigated angles are provided in Figure S1a. Because the membrane is fragile during transfer, preparing multiple samples with exactly the same target angle, especially below $10^{\circ }$, remains technically challenging in the present process.

**FIGURE 1:**
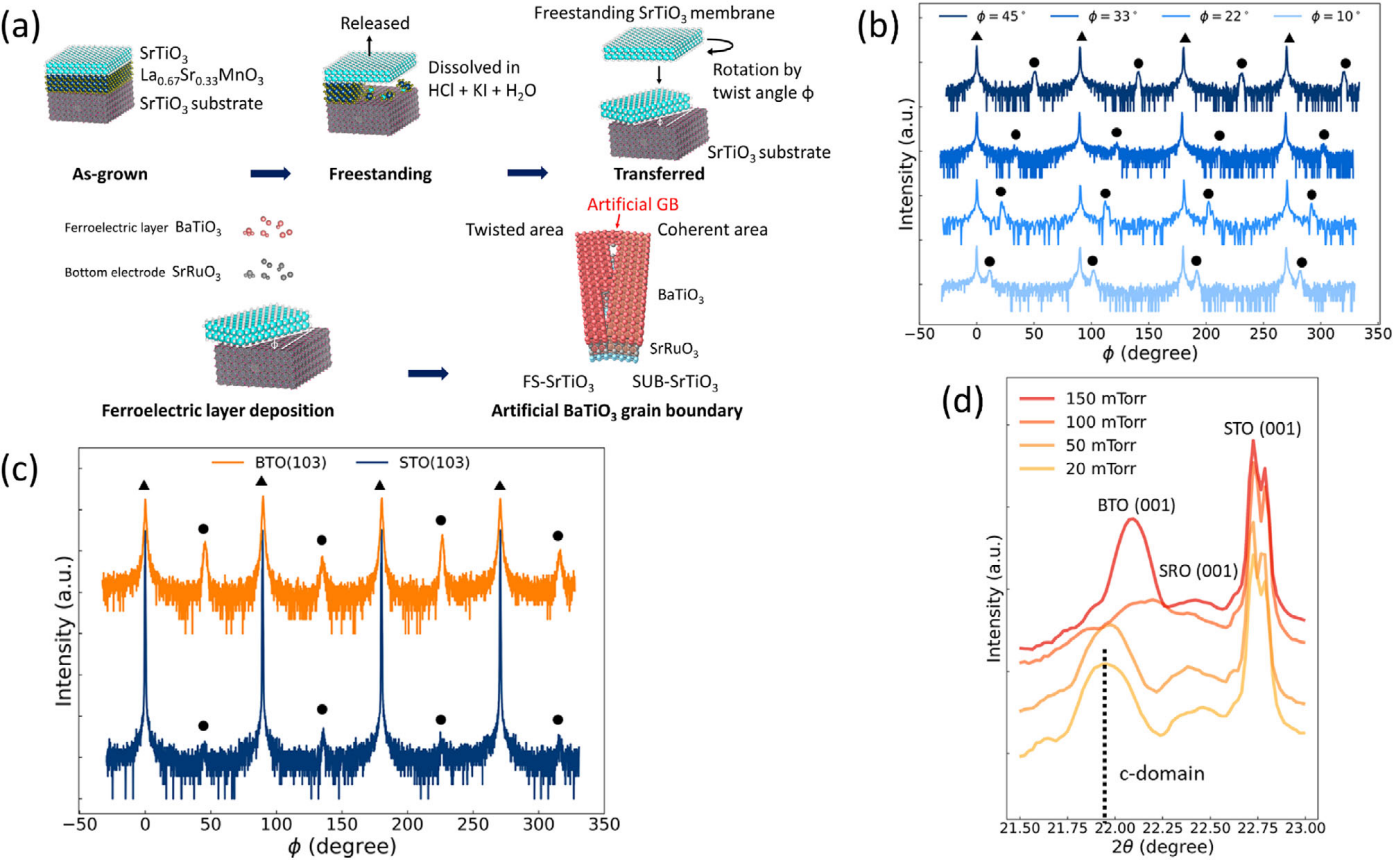
(a) Schematic illustration of the artificial‐grain‐boundary fabrication process, explicitly showing the FS‐STO, SUB‐STO, artificial GB, coherent area (CA), twisted area (TA), bottom SRO electrode, and BTO ferroelectric layer. (b) Phi scans of substrate STO(103) (triangles) and freestanding STO(103) (circles) with misorientations from $10^{\circ }$ to $45^{\circ }$. (c) Phi scans of substrate STO(103)/coherent BTO(103) (triangles) and freestanding STO(103)/twisted BTO(103) (circles). (d) θ–2θ scans of BTO(001) grown under different oxygen pressures. The dashed line labels the corresponding 2θ position of the c‐domain.

Next, a BTO layer and a bottom ${\mathrm{SrRuO}}_{3}$ (SRO) electrode were grown on the substrate with a well‐defined grain boundary. The resulting tilted grain boundary is illustrated schematically in Figure 1a, and a corresponding bright‐field TEM image from a preliminary structural‐confirmation sample is provided in Figure S1b to verify the artificial grain‐boundary geometry. In this study, the BTO region grown on the single‐crystalline STO side (SUB‐STO) is defined as the coherent area (CA), whereas the BTO region grown on the freestanding rotated STO side (FS‐STO) is defined as the twisted area (TA). As shown in Figure 1c, the BTO (103) reflections coincide with the STO (103) reflections, confirming the successful fabrication of the artificial grain boundary structure.

BTO films were deposited under oxygen pressures ranging from 20 to 150 mTorr, all maintaining a pure tetragonal phase (Figure 1d). As oxygen pressure decreases, the BTO (001) peak shifts toward smaller 2θ angles, signaling an a‐domain to c‐domain transition, which is consistent with the trend reported in previous studies [25]. We selected 20mTorr for further investigation, as the resulting c‐domain structure maximizes out‐of‐plane ferroelectricity through enhanced *c*‐axis atomic displacement [26]. Reciprocal space mappings (RSMs) and calculated lattice constants (Figure S1c) show that BTO maintains identical lattice parameters across the grain boundary, ensuring that any variation in ferroelectric properties is not due to crystallographic differences. Finally, 20 nm BTO (with angles of $26^{\circ }$, $34^{\circ }$, and $45^{\circ }$) and 50nm BTO (with angles of $10^{\circ }$, $22^{\circ }$, and $45^{\circ }$) were characterized for their macroscopic polarization, domain dynamics, and leakage performance. The different angle sets reflect the experimentally accessible artificial GB samples produced by the freestanding‐transfer process. In the present study, the 20nm films were used mainly for quantitative angle‐dependent leakage modeling, whereas the 50nm films were used to examine whether GB‐localized leakage persists when the BTO thickness is increased.

## 3 Results and Discussion

### 3.1 Macroscopic Ferroelectric Response

To evaluate the macroscopic ferroelectric properties, polarization‐voltage (P–V) and current‐voltage (I–V) characteristics were measured across the CA, TA, and the GB area situated at their junction (Figure 2a). The optical microscopy image of the GB area is provided in Figure S2a. The P–V loop from the CA exhibits saturated polarization ($P_{s}$) and coercive voltage ($V_{c}$) typical of high‐quality BTO, thereby validating the structural and functional integrity of the epitaxial layers prior to grain boundary analysis [27]. In contrast, the TA shows greater leakage and a higher $V_{c}$ than the CA. This is attributed to a twisted GB at the FS‐STO/SUB‐STO interface, where a high density of interfacial defects propagates into the BTO layer during high‐temperature epitaxial growth, thereby degrading ferroelectric performance in the TA [28].

**FIGURE 2:**
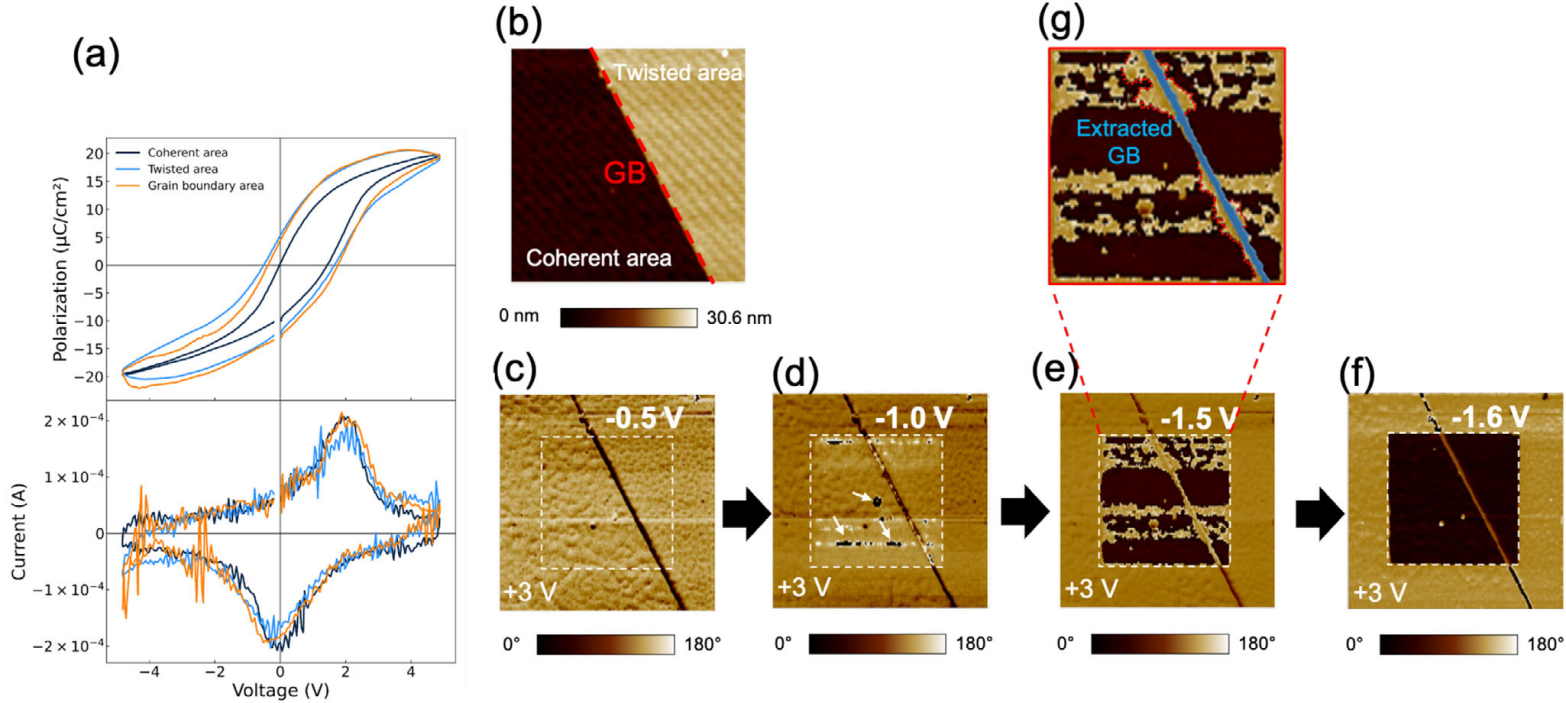
(a) Ferroelectric polarization–voltage (P–V) and current–voltage (I–V) hysteresis loops measured at the coherent area (CA), twisted area (TA), and grain boundary (GB) region under a maximum applied voltage of 5V. (b) Surface topography around the grain boundary. Out‐of‐plane PFM phase images after poling with +3V over a 5μm × 5μm area, followed by reversed bias applied over a central 3μm × 3μm area at (c) −0.5V, (d) −1V, (e) −1.5V, and (f) −1.6V. (g) Magnified view of the grain boundary region in (e); the solid blue line marks the position of the GB, and the red dashed lines highlight domain wall pinning at the GB. White arrows in (d) indicate sites of domain nucleation.

The evolution of P–V and I–V curves under increasing maximum bias (from 3 to 5 V) illustrates the field‐dependent response of the BTO films (Figure S2b,c). While the CA and TA exhibit similar $P_{s}$ and $V_{c}$ values at a low bias of 3 V, a clear divergence emerges at higher voltages. This suggests that higher electric fields activate the dense defect networks in the BTO ferroelectric layer, thereby increasing leakage current. This field‐induced leakage integrates non‐switching current components into the measurement, leading to the characteristic rounding of the P–V loop tails and an artificial enhancement of the $P_{s}$ [29].

Finally, the GB area, which encompasses the CA, TA, and the tilted grain boundary between them, exhibits an electrical response representing a composite of all three regions. Notably, the $V_{c}$ of the GB area is significantly larger than the arithmetic average of the CA and TA, and is more comparable to the TA value. This indicates that a high density of defects is localized at the BTO tilted boundary. These defects likely act as strong electronic traps and pinning centers in BTO, simultaneously increasing leakage current and hindering domain switching [30]. Given that the macroscopic ferroelectric properties are integrated across these three regions and that defects are predominantly activated at higher voltages, a further investigation of domain wall switching was conducted at a relatively low bias of 3 V to isolate the intrinsic switching behavior.

### 3.2 Switching Dynamics Near the Grain Boundary

To gain insight into the switching dynamics, including domain nucleation and propagation near the GB area, we performed out‐of‐plane PFM across a region containing the CA, TA, and GB, as shown in Figure 2b. Topography, phase, and amplitude images specifically for the CA and TA regions are provided in Figure S3a [22]. It should be noted that weak stripe‐like contrast on the topography of TA (Figure S3a). However, FFT analysis indicates that this periodic feature is caused by instrumental vibration rather than by an intrinsic surface morphology (see Figure S9). Therefore, the topography image is used here mainly to identify the grain‐boundary region for comparison with the local current map. Initially, a positive DC bias of +3V was applied over a 5μm × 5μm area to achieve uniform polarization alignment. This voltage was specifically chosen based on the P–V loop results to minimize leakage contributions originating from voltage‐activated defects. Subsequently, a series of incremental negative DC biases (−0.5V to −1.6V) was applied to a central 3μm × 3μm area to induce polarization reversal as shown in Figure 2c–f. To highlight the domain dynamics, the analysis focuses on phase images with phase shifts to 180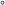, while the original phase and amplitude data are provided in Figure S3b.

At a reverse bias of −0.5V, the phase image remains featureless, showing no detectable contrast (Figure 2c). As the bias increases to −1.0V, isolated dark‐colored domains nucleate within both the CA and TA, marking the onset of ferroelectric switching (Figure 2d). Upon further increasing the bias to −1.5V, these domains expand laterally toward the GB; however, their propagation is strongly arrested near the interface, where domain wall motion is effectively pinned by localized defects (Figure 2e) [18, 21].

To decouple intrinsic domain behavior from topographic artifacts, the GB topography from the −0.5V scan is extracted and overlaid onto the −1.5V phase image (Figure 2e). The magnified view in Figure 2g reveals a clear spatial discrepancy between the domain boundaries and the grain boundary morphology. Specifically, the domain walls exhibit a distorted, non‐linear profile that fails to align with the straight topographic steps of the GB (Figure 2c) [20]. These irregular domain shapes confirm that domain growth is inhibited by pinning centers localized at the boundary. Crucially, point‐and‐shoot piezoresponse hysteresis loops confirm the presence of intrinsic ferroelectricity directly at the GB (Figure S3c) [22]. Finally, a reverse bias of −1.6V achieves full polarization reversal within the scanned area (Figure 2f). These microscopic PFM observations align perfectly with our macroscopic findings: the structural disorder and elevated defect density at the tilted interface create a significant energy barrier to domain wall motion, thereby increasing the local coercive field and modulating the switching dynamics at the artificial boundary [17, 21].

### 3.3 Angle‐Dependent Leakage

The local leakage behavior at the artificial grain boundary was first examined by c‐AFM, as summarized in Figure 3. Figure 3a shows the surface topography in the grain‐boundary region. The corresponding c‐AFM result is shown in Figure 3b. In strong contrast to the surrounding CA and TA, which remain highly insulating, a pronounced current signal is localized along the artificial grain boundary. The current line profile taken across the boundary further confirms that the conduction is strongly concentrated within a narrow region centered at the GB. This result demonstrates that the artificial GB acts as a localized low‐resistance leakage pathway rather than a uniformly distributed leakage source over the entire capacitor area.

**FIGURE 3:**
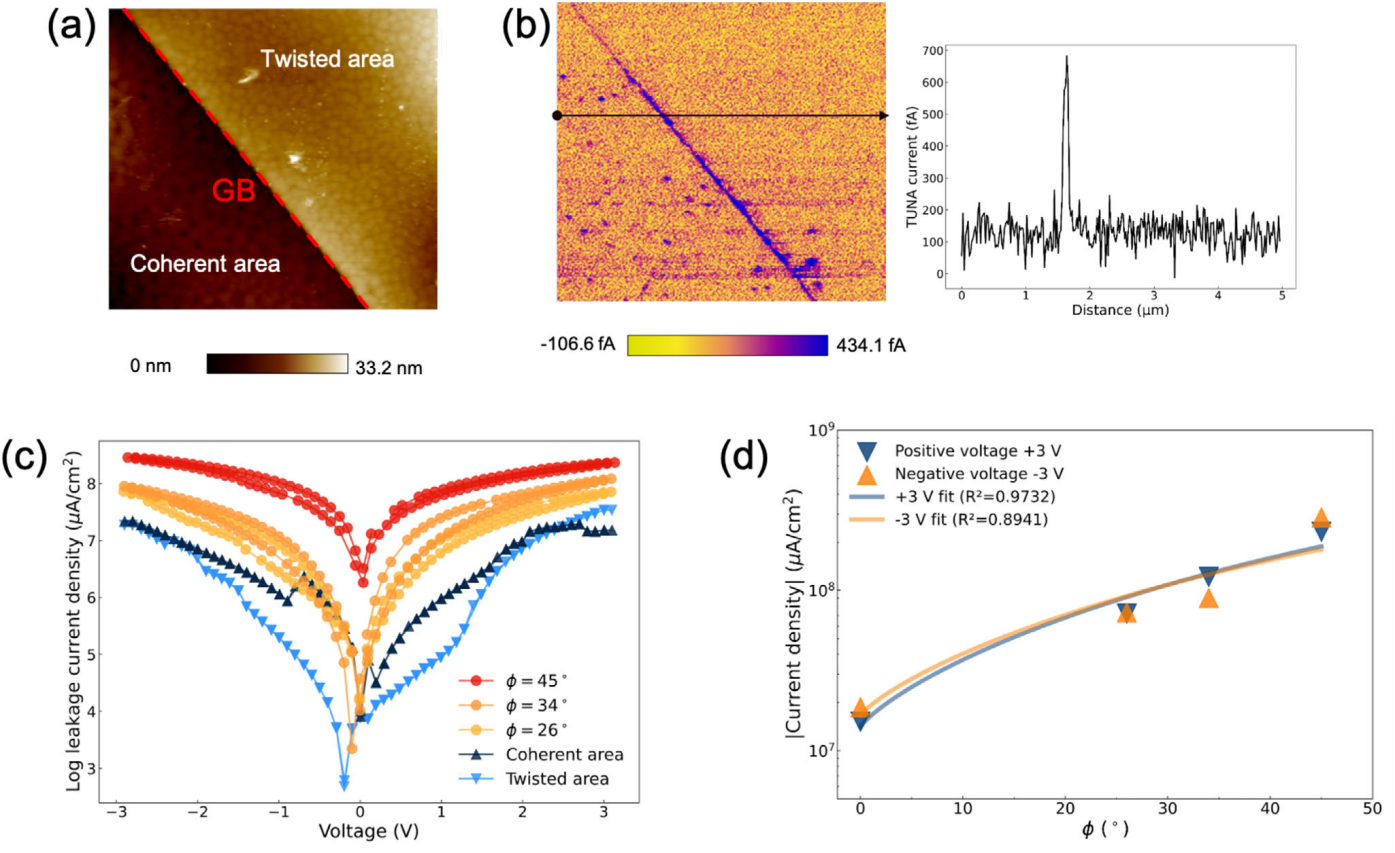
(a) Surface topography at the GB region. (b) Local current mapping and the corresponding current line profile across the GB, showing that the leakage current is strongly localized along the boundary. (c) Logarithm of the leakage current density for the 20nm BTO film at the coherent area (CA), twisted area (TA), and for misorientation angles of $26^{\circ }$, $34^{\circ }$, and $45^{\circ }$. (d) Extracted leakage current at +3V as a function of misorientation angle, fitted with the Read–Shockley‐inspired leakage model.

To quantify the leakage behavior, the current density measured at +3V is compared in Figure 3c for the CA, TA, and the grain‐boundary devices with different misorientation angles in the 20nm BTO films. The CA and TA show comparatively low baseline leakage, whereas the GB devices exhibit substantially larger current density. Moreover, the leakage current increases systematically with increasing grain‐boundary misorientation angle from $26^{\circ }$ to $45^{\circ }$. This trend indicates that the GB‐related leakage contribution becomes stronger as the crystallographic mismatch across the boundary increases.

The angle dependence is summarized more explicitly in Figure 3d, where the extracted leakage current at +3V is plotted as a function of misorientation angle and fitted using the Read–Shockley‐inspired leakage model discussed in the next subsection. The measured current shows a clear monotonic increase with angle and is well described by the fitting function for the 20nm BTO films. Physically, this behavior is consistent with the view that increasing misorientation enhances the effective structural disorder at the grain boundary, thereby increasing the density of transport‐active defect or trap states and lowering the effective barrier for leakage conduction.

We note that a similar GB‐localized leakage trend is also observed in the 50nm BTO films (see Figure S4a,d), indicating that the presence of a localized conductive GB pathway is not limited to the thinner films. However, the quantitative fitting in Figure 3d is focused on the 20nm BTO films, for which the GB contribution is more clearly resolved. The thicker films may involve additional thickness‐dependent effects, including strain relaxation, domain configuration, vertical transport length, and the relative competition between GB conduction and bulk leakage. Therefore, while the persistence of GB‐localized leakage is confirmed in both thicknesses, a complete thickness‐dependent scaling analysis remains beyond the scope of the present work.

### 3.4 Defect‐Assisted Leakage Model

The leakage current density (J) was modeled as a function of the misorientation angle (ϕ) using a Read–Shockley‐inspired framework derived from dislocation theory [1]. In the low‐angle limit, the grain boundary can be described as an array of discrete dislocations, for which the effective structural‐disorder term follows

(1) $$\rho_{\mathrm{eff}}\left(\phi \right)\propto \phi \left(A-\ln\phi \right),$$

where A is a phenomenological parameter related to the localized energy of atomic misfit [31]. In the present work, $\rho_{\mathrm{eff}}\left(\phi \right)$ should be understood as an *effective* transport‐active defect/trap‐state density rather than a directly measured microscopic defect concentration. As the misorientation angle increases, the spacing between neighboring dislocations decreases and the overlap of dislocation cores and strain fields is expected to increase, leading to a more structurally disordered interface. Possible microscopic contributors to the transport‐active states include dislocation‐core states, oxygen‐vacancy‐related states, strain‐induced defect complexes, cation disorder, and antisite defects. However, the present electrical measurements do not directly identify the specific defect species or their detailed energy distribution.

Within this phenomenological picture, the leakage follows a thermally activated form,

(2) $$J\propto \exp\left(-\frac{E_{a}}{k_{B}T}\right),$$

where J is the current density, $E_{a}$ is the effective activation barrier for charge transport, $k_{B}$ is the Boltzmann constant, and T is the absolute temperature. By combining the structural‐disorder term with the thermally activated transport law, the total leakage current density is expressed as

(3) $$J\left(\phi \right)=J_{bg}+J_{0}\exp\left[\beta \cdot \phi \left(A-\ln\phi \right)\right],$$

where $J_{bg}$ is the background leakage of the coherent area and β is a coupling coefficient representing the sensitivity of the transport to angle‐dependent structural disorder.

As shown in Figure 3d, this integrated model successfully captures the strong increase in leakage current observed in the 20nm BTO films, with $R^{2}$ values of 0.9732 and 0.8941 for the positive and negative branches, respectively. We emphasize that the Read–Shockley‐inspired term is used here as a phenomenological descriptor of the angle dependence of structural disorder and transport‐active defect/trap states, rather than as a direct measurement of microscopic defect density.

### 3.5 Thermally Activated Transport

To further examine the transport behavior of the grain‐boundary region, temperature‐dependent I–V measurements were performed on the CA, TA, and GB areas over a temperature range of 20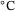 – 100 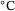. The transport analysis was based on *steady‐state DC leakage current* measured at a fixed bias of +3V after the transient charging current had decayed, rather than on AC impedance or equivalent‐circuit fitting. In all regions, the leakage current increases with temperature, indicating thermally activated transport.

As discussed in the P–V loop analysis, the GB area under a single top electrode inherently includes contributions from the adjacent CA and TA. Consequently, the measured conduction corresponds to an effective parallel configuration involving the CA, TA, and GB. At each temperature, the resistance of each region was defined from the stabilized DC leakage current using Ohm's law,

(4) $$R\left(T\right)=\frac{V}{I(T)},$$

with V=+3V. To extract the intrinsic GB contribution, the measured conduction was analyzed using the parallel‐resistance relation

(5) $$\frac{1}{R_{\mathrm{parallel}}}=\frac{1}{R_{\mathrm{CA}}}+\frac{1}{R_{\mathrm{TA}}}+\frac{1}{R_{\mathrm{GB}}},$$

where $R_{\mathrm{parallel}}$ is the experimentally measured resistance of the GB‐area capacitor and $R_{\mathrm{GB}}$ is the extracted grain‐boundary resistance. The effective parallel‐resistance configuration and the temperature‐dependent steady‐state DC leakage traces used for this extraction are provided in Figure S5.

Figure 4b shows the temperature dependence of the resistance for each component. For clarity, the legend “Grain boundary area (in parallel)” refers to the experimentally measured parallel configuration, whereas “Grain boundary (extracted)” denotes the GB contribution isolated from Equation (5). The extracted values reveal that $R_{\mathrm{GB}}$ is comparable to the total $R_{\mathrm{parallel}}$, confirming that the GB acts as the dominant conduction pathway within the GB area.

**FIGURE 4:**
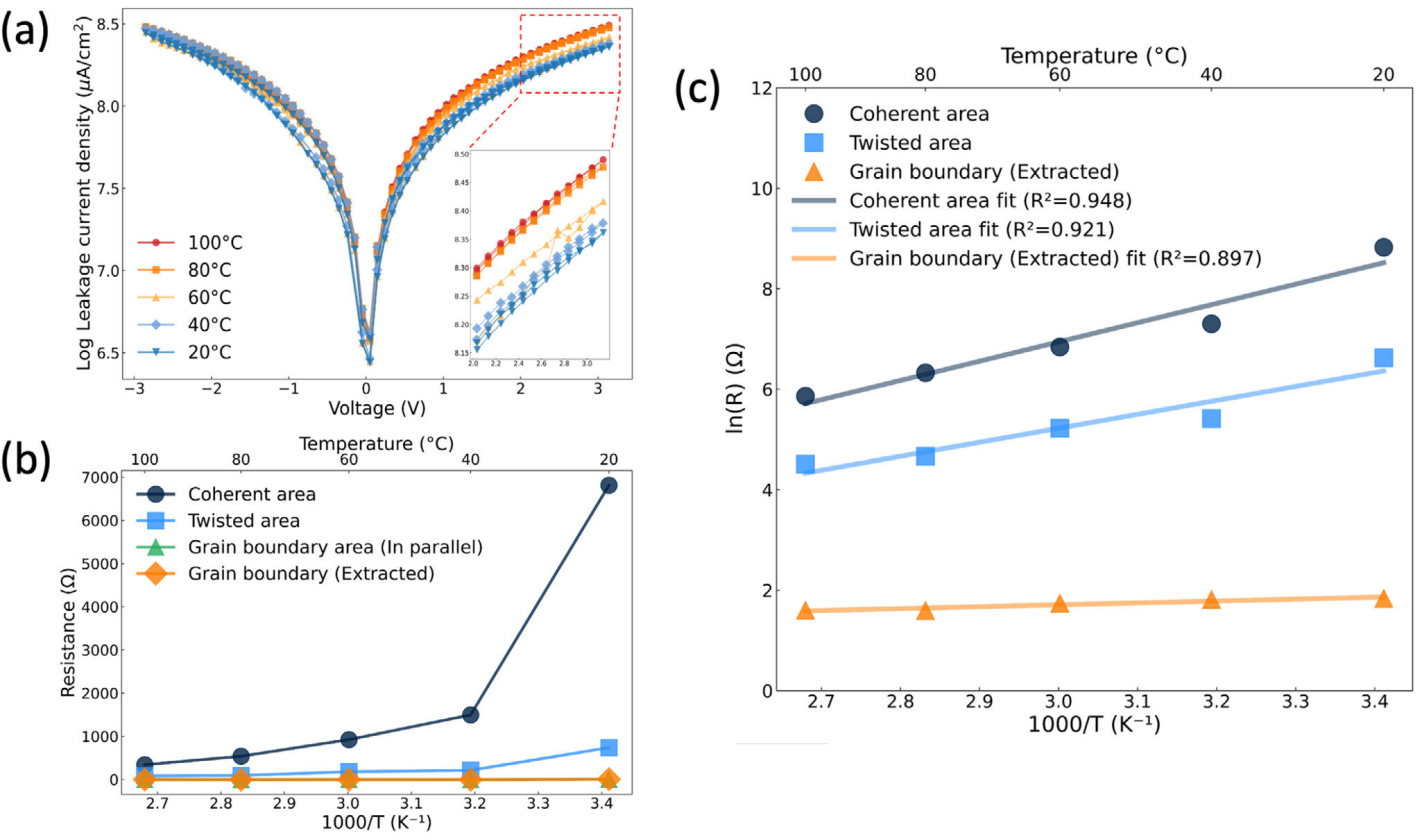
(a) Temperature‐dependent steady‐state DC leakage current of 20nm BTO with misorientation angle $45^{\circ }$. (b) Resistance of the CA, TA, measured grain‐boundary area (in parallel), and extracted GB as a function of temperature, where each resistance was calculated from the stabilized current at +3V using Ohm's law. (c) Arrhenius plots of lnR versus 1/T for the CA, TA, and extracted GB. Solid lines are linear least‐squares fits used to determine the activation energies, and the fitting uncertainty was obtained from the standard error of the slope. The steady‐state DC leakage traces used for the resistance extraction are shown in Figure S5, and the Schottky and Poole–Frenkel comparisons are summarized in Figures S6–S8.

The activation energy was obtained from Arrhenius plots of lnR versus 1/T, as shown in Figure 4c, using

(6) $$\ln R=\ln R_{0}+\frac{E_{a}}{k_{B}T}.$$

Linear least‐squares fitting was used to determine the slope, and the uncertainty in $E_{a}$ was evaluated from the standard error of the fitted slope. The extracted $E_{a}$ values are 0.332eV and 0.239eV for the CA and TA, respectively, whereas the GB exhibits a substantially lower value of 0.032eV, nearly an order of magnitude smaller than the surrounding regions. The much smaller activation barrier at the GB is consistent with its enhanced local conductivity.

To further distinguish the leakage mechanism, we compared Schottky and Poole–Frenkel linearizations using the same positive‐bias upsweep dataset for the 20nm BTO grain‐boundary capacitor. The Schottky plots, direct Schottky‐versus‐Poole–Frenkel comparison, and summary of the fitted $R^{2}$ values are provided in Figures S6–S8, respectively. The Schottky representation, $\ln(J/T^{2})$ versus $E^{1/2}$, consistently gave substantially better linearity than the Poole–Frenkel representation, ln(J/E) versus $E^{1/2}$, over the selected field range. These results indicate that the measured leakage is more consistent with defect‐assisted, Schottky‐like thermally activated emission than with bulk Poole–Frenkel conduction. In addition, the strong electrical contrast between the grain boundary and the surrounding grains may give rise to local interfacial polarization or Maxwell–Wagner‐type charge accumulation under an applied field, a picture that has also been discussed for grain‐boundary‐mediated electrical response in ferroelectric ceramic systems [32]. Therefore, the low activation energy at the GB is interpreted as evidence of a strongly reduced effective carrier‐injection barrier caused by defect‐rich structural disorder, rather than as stand‐alone proof of pure hopping conduction.

Notably, the activation energy at the GB is comparable to the thermal energy at room temperature ($k_{B}T∼0.026\;\mathrm{eV}$ at 300 K), which explains the weak temperature dependence of the GB resistance. Collectively, these results show that the artificial grain boundary introduces a low‐barrier conduction pathway that dominates the macroscopic leakage behavior.

## 4 Conclusion

We create artificial grain boundaries in ferroelectric BTO using a freestanding membrane‐transfer method and investigate their influence on ferroelectric switching and leakage through combined macroscopic and microscopic characterization. Ferroelectric P–V measurements reveal that both the coercive voltage and leakage current are enhanced in the GB region.

PFM switching measurements show that domain‐wall propagation is hindered near the artificial GB, consistent with propagation‐limited switching and the increased coercive voltage. C‐AFM measurements further demonstrate a pronounced current response localized at the GB, identifying the boundary as a dominant local leakage pathway. The angle‐dependent leakage measurements reveal a strong increase in current with increasing GB angle, consistent with a Read–Shockley‐inspired description in which increasing misorientation enhances the effective structural disorder and transport‐active defect/trap‐state density.

Temperature‐dependent steady‐state DC leakage analysis shows that the activation energy at the GB is approximately one order of magnitude smaller than that in the coherent area and is close to the thermal energy at room temperature. Together with the direct comparison between Schottky and Poole–Frenkel analyses, these results indicate that the leakage is more consistent with defect‐assisted, Schottky‐like thermally activated emission than with bulk Poole–Frenkel conduction. The grain boundary is therefore interpreted as a defect‐rich region that lowers and spatially inhomogenizes the effective carrier‐injection barrier.

Finally, we emphasize that the present work focuses on the initial GB‐induced switching and leakage behavior. A complete understanding of the microscopic defect structure, long‐term reliability under repeated electrical stress, and thickness‐dependent scaling of GB contributions will require future atomic‐resolution structural characterization and systematic electrical studies. Other GB‐related properties, such as resistive switching and photovoltaic effects, bias‐ and time‐dependent domain dynamics, and the retention behavior of domain switching, remain to be explored. Furthermore, the freestanding method proposed here for artificial grain boundary fabrication may be further extended to other oxide material systems, such as perovskite ferroelectrics or ${\mathrm{HfO}}_{2}$‐based ferroelectric oxides, provided that a suitable sacrificial layer, release process, and mechanically stable freestanding membrane structure can be established.

## 5 Experimental Sections

### 5.1 Samples Preparation

The heterostructures were synthesized on (001)‐oriented ${\mathrm{SrTiO}}_{3}$ (STO) substrates via pulsed‐laser deposition (PLD) using a KrF excimer laser with the wavelength 248nm. ${\mathrm{La}}_{0.67}$ ${\mathrm{Sr}}_{0.33}$ ${\mathrm{MnO}}_{3}$ (LSMO) and ${\mathrm{SrRuO}}_{3}$ (SRO) sacrificial/bottom electrode layers were grown at $750^{\circ }C$ ($2.5\times 10^{-1}\;\mathrm{Torr}$) and $700^{\circ }C$ ($5\times 10^{-2}\;\mathrm{Torr}$), respectively, followed by the deposition of the ferroelectric ${\mathrm{BaTiO}}_{3}$ (BTO) layer at $700^{\circ }C$ and $2.1\times 10^{-2}\;\mathrm{Torr}$. Throughout the process, the laser fluence was maintained at $1.3\;Jcm^{-2}$ with a target‐to‐substrate distance of 5.5cm. Repetition rates were set at 10 Hz for LSMO, 5 Hz for SRO, and 2 Hz for the BTO. Finally, the samples were cooled to room temperature at $10^{\circ }Cmin^{-1}$ under an oxygen pressure of $4.5\times 10^{2}\;\mathrm{Torr}$ to ensure proper stoichiometry.

### 5.2 Freestanding Process

A solution of 0.1 g KI + 10 mL HCl + 100 mL deionized water was used to dissolve the LSMO sacrificial layer at room temperature. The floating STO membrane is transferred directly onto a STO substrate without a protective/supporting layer.

### 5.3 Capacitor Fabrication

Top electrodes, with a thickness of 30 nm, were patterned into circular structures (diameter = 10 to 100μm) via a standard lift‐off photolithography process. The metal deposition was performed using PLD at room temperature under an argon ambient of $3.0\times 10^{-2}$ Torr.

### 5.4 X‐Ray Scattering

Structural characterization of the heterostructures was performed using high‐resolution X‐ray diffraction (HRXRD, Bruker D8) with a Cu $K\alpha_{1}$ radiation source (λ=1.5406 Å). The phase purity and crystal orientation were confirmed via θ–2θ scans, while the epitaxial relationship and four‐fold symmetry of the BTO/STO layers were verified through phi scans. To quantify the lattice parameters and evaluate the epitaxial strain state, reciprocal space mappings (RSMs) was conducted. Additionally, X‐ray reflectivity (XRR) measurements were employed to precisely determine the film thickness and assess the interfacial roughness.

### 5.5 Ferroelectric Properties Measurement

Electrical characterizations were conducted in a metal–ferroelectric–metal (MFM) capacitor configuration using an aixACCT TF‐3000 analyzer. Ferroelectric polarization–voltage (P–V) hysteresis loops were recorded using a triangular waveform at frequencies ranging from 1 to 10 kHz. Capacitance–voltage (C–V) characteristics were used only as a preliminary quality check for the single‐layer BTO films and were not used for the grain‐boundary resistance extraction or activation‐energy analysis. Leakage current was measured from −3 to +3 V with a 0.1 or 0.5 V increment. Temperature‐dependent transport analysis was performed using steady‐state DC leakage current measured at a fixed bias of +3V after the transient charging current had decayed, with data collected at each stabilized temperature step from $20^{\circ }C$ to $100^{\circ }C$.

### 5.6 Scanning Probe Microscopy

A Bruker Dimension Icon system was utilized for simultaneous PFM and c‐AFM analyses in contact mode. Conductive PtIr‐coated probes were used for both measurements, with a nominal spring constant range of 1.2–29 N/m. The PFM images were acquired with a scan rate of 2 Hz and a pixel resolution of 256 samples per line 128 lines. To induce and image local polarization states, box‐in‐box lithography was executed by applying a ±3V bias to the tip while the bottom electrode was grounded. The local ferroelectricity of the films was further validated via point‐and‐shoot spectroscopy (switching spectroscopy PFM), which recorded the voltage‐dependent phase and amplitude signals. The complete bias waveform contained 256 voltage steps. The dwell time per step was controlled by the instrument acquisition settings. For c‐AFM measurement, the contact setpoint was set to 0.07 V in the instrument feedback signal, and a DC bias of 10 V was applied during current mapping.

## Author Contributions

Y.‐L.H. designed and supervised the study. S.‐R.Y. wrote the manuscript. Y.‐G.W. and Y.‐C.F. grew the materials, fabricated ferroelectric devices, and performed the ferroelectric measurements. Y.‐G.W. and H.‐J.L. conducted measurements of XRD. Y.‐G.W., Y.‐T.L., and Y.‐C.C. conducted the AFM and PFM measurements. C.‐C.W. and J.‐C.Y. aided in the freestanding process. Y.‐C.S., Y.‐W.C., and Y.‐H.L. aided in the ferroelectric measurements. C.‐Y.C. aided in the XRD measurement and analysis in NSRRC. Y.‐L.H. and all the authors made contributions to writing the manuscript.

## Conflicts of Interest

The authors declare no conflicts of interest.

## Supporting information


**Supporting File**: advs77239‐sup‐0001‐SuppMat.docx.

## Data Availability

The data that support the findings of this study are available from the corresponding author upon reasonable request.
