# Supplementary material for: Artificial Grain Boundaries Created by Freestanding Membrane Transfer Reveal Angle‐Dependent Ferroelectric Switching and Leakage in BaTiO3
Source: Adv Sci (Weinh). 2026 Oct 14:e77239. Online ahead of print. doi: 10.1002/advs.77239 (PMC13667410; doi:10.1002/advs.77239)
Supplement: Supplementary file 1 [file ADVS-9999-e77239-s001.docx]

**Supporting information of Artificial Grain Boundaries Created by Freestanding Membrane Transfer Reveal Angle-Dependent Ferroelectric Switching and Leakage in BaTiO_3_**

Shin-Rou Yin^1^, Yun-Guan Wei^1^, Yu-Cheng Fang^1^, Yung-Chi Su^1^, Yu-Wei Chen^1^, Yu-Hsin Lin^1^, Yu-Ting Lin^2^, Chia-Chun Wei^2^, Heng-Jui Liu^3^, Ching-Yu Chiang^4^, Jan-Chi Yang^2^, Yi-Chun Chen^2^, Yen-Lin Huang^1*^

1. *Department of Materials Science and Engineering, National Yang Ming Chiao Tung University, Hsinchu, Taiwan.*
2. *Department of Physics, National Cheng Kung University, Tainan, Taiwan.*
3. *Department of Materials Science and Engineering, National Chung Hsing University, Taichung, Taiwan.*
4. *National Synchrotron Radiation Research Center, Hsinchu, Taiwan.*

Fig. S1 (a) Phi scans of substrate STO(103) (triangles) and freestanding STO(103) (circles) with misorientations from 26°, 34°, and 45°. (b) Bright-field TEM image of an early-stage structural-confirmation sample showing the artificial grain boundary (red circle) formed via the 10-nm STO freestanding membrane (yellow circle). This image is used to confirm the existence and geometry of the artificial grain boundary; the final electrical, PFM, and c-AFM measurements were performed on the optimized SRO-based device structure. (c) RSMs of STO (103) of CA and TA, and the calculated lattice constants.

Fig. S2 (a) Optical microscopy image of the capacitor, with the artificial grain boundary indicated by a yellow dashed line. Ferroelectric P–V and I–V measurements at (b) CA and (c) TA under applied voltages from 3 V to 5 V.

Fig. S3 (a) PFM topography, phase, and amplitude images of the CA and TA regions. PFM poling was performed with +5 V over a 5 μm × 5 μm area and −3 V over a central 3 μm × 3 μm area. (b) PFM phase and amplitude images of the GB area after poling with +3 V over a 5 μm × 5 μm area, followed by −0.1, −0.5, −1.0, and −1.6 V over a central 3 μm × 3 μm area. The stripe-like contrast in the topography is attributed to instrumental vibration rather than intrinsic surface morphology, and the phase contrast exactly at the GB may be influenced by the kink-like topography introduced by the transferred 10-nm STO membrane. (c) PFM point-and-shoot phase–voltage hysteresis loops measured across the GB region, confirming local switchable ferroelectric response.

Fig. S4 (a) Conductive AFM current mapping of 50-nm-thick BTO, showing grain-boundary-localized conduction. (b) Logarithm of leakage current measurement of 50-nm BTO at the CA, TA, and misorientation angles of 10°, 22°, and 45°. The extracted leakage current at +3 V and −3 V of (c) 20-nm BTO and (d) 50-nm BTO is plotted as a function of misorientation angle. Both thicknesses show increasing leakage with increasing grain-boundary angle, while the quantitative Read–Shockley-inspired analysis is mainly applied to the 20-nm BTO films. The lower background leakage in the 50-nm films suggests that thickness-dependent effects may modulate the quantitative GB contribution.

Fig. S5 (a) Schematic illustration of the effective parallel-resistance configuration used to extract the intrinsic grain-boundary resistance from the CA, TA, and GB-area measurements. Temperature-dependent steady-state DC leakage current measured at +3 V after transient charging had decayed at (b) CA and (c) TA. These DC leakage measurements, rather than AC impedance or C–V analysis, were used for the resistance extraction and Arrhenius fitting.

Fig. S6 Schottky emission analysis of temperature-dependent leakage current. Linearized Schottky plots of the positive-bias leakage data for the 20-nm BTO capacitor at temperatures from 20 to 100 °C, presented as ln(J/T^2^) versus E^1/2^, where J is the current density, T is the absolute temperature, and E is the electric field across the film. Symbols represent the experimental data and the red lines are linear fits. The extracted R^2^ values indicate that the Schottky representation provides a good description of the temperature-dependent leakage behavior over the selected field range.

Fig. S7 Direct comparison between Schottky and Poole–Frenkel analyses using the same temperature-dependent positive-bias leakage dataset. The Schottky linearization shows consistently higher linearity than the Poole–Frenkel linearization, supporting a defect-assisted, Schottky-like leakage interpretation.

Fig. S8 Summary of R² values obtained from the Schottky and Poole–Frenkel fits at different temperatures. The systematically higher R² values of the Schottky fits indicate that the leakage current is more consistent with Schottky-like emission than with Poole–Frenkel conduction.

Fig. S9 FFT-based removal of periodic stripe noise in the AFM image. (a) Original AFM topography image showing a pronounced diagonal periodic stripe artifact. (b) Two-dimensional fast Fourier transform (FFT) magnitude spectrum of the original image, where the strongest off-center peak pair associated with the periodic stripe noise is marked. (c) AFM image reconstructed after applying a notch filter to remove only the dominant FFT peak pair, suppressing the periodic stripe artifact while preserving the main topographic features.
